# Three-Dimensional Extended Target Tracking and Shape Learning Based on Double Fourier Series and Expectation Maximization

**DOI:** 10.3390/s25154671

**Published:** 2025-07-28

**Authors:** Hongge Mao, Xiaojun Yang

**Affiliations:** 1 School of Information Engineering, Chang’an University, Xi’an 710064, China; maohongge@nynu.edu.cn; 2 School of Artificial Intelligence and Software Engineering, Nanyang Normal University, Nanyang 473061, China

**Keywords:** target tracking, shape estimation, expectation conditional maximization, double Fourier series, axis-angle

## Abstract

This paper investigates the problem of tracking targets with unknown but fixed 3D star-convex shapes using point cloud measurements. While existing methods typically model shape parameters as random variables evolving according to predefined prior models, this evolution process is often unknown in practice. We propose a particular approach within the Expectation Conditional Maximization (ECM) framework that circumvents this limitation by treating shape-defining quantities as parameters estimated directly via optimization. The objective is the joint estimation of target kinematics, extent, and orientation in 3D space. Specifically, the 3D shape is modeled using a radial function estimated via double Fourier series (DFS) expansion, and orientation is represented using the compact, singularity-free axis-angle method. The ECM algorithm facilitates this joint estimation: an Unscented Kalman Smoother infers kinematics in the E-step, while the M-step estimates DFS shape parameters and rotation angles by minimizing regularized cost functions, promoting robustness and smoothness. The effectiveness of the proposed algorithm is substantiated through two experimental evaluations.

## 1. Introduction

The accurate perception and tracking of targets in three-dimensional (3D) environments are a fundamental requirement for many modern intelligent systems, including autonomous vehicles, robotic platforms, and advanced surveillance systems [1,2,3,4]. While traditional point target tracking algorithms assume that one target only gives rise to one measurement, they are insufficient when dealing with objects possessing significant spatial extent relative to sensor resolution, known as extended target [5,6]. The effective tracking of these extended targets demands algorithms that can estimate not only their kinematic state (position, velocity) but also their physical characteristics, including shape and orientation, within a 3D space.

A substantial body of work considers the extended target tracking (ETT) problem, especially in 2D space [5,6]. Corresponding methods process 2D measurements based on diverse extent models. One common approach employs random matrix to model the target’s shape as an ellipse [7,8]. These extent models often assume that the extent state follows a specific probability distribution, such as a Gaussian distribution. However, this underlying distribution is frequently unknow in practice. Addressing this limitation, paper [9] presents an alternative method where extent characteristics, including semi-axis lengths, are treated as parameters for direct estimation. For complex star-convex targets, more sophisticated shape modeling methods, such as B-splines [10] and Gaussian processes (GPs) [11,12], have been developed. Notably, the random hyper-surface model (RHM) is a typical approach for representing arbitrary star-convex shapes within the frequency domain [13,14]. This model defines the target’s spatial extent using a Fourier series expansion, with its coefficients jointly estimated alongside the target’s kinematics. RHM offers a parameterization particularly well-suited for star-convex extents, effectively balancing representational accuracy with computational complexity. Numerous adaptations of such Fourier-based approaches have subsequently been explored across diverse application settings [15,16,17,18,19,20,21].

When 3D surface measurements from devices like multilayer LiDAR sensor or depth cameras are available, it becomes possible to model the target’s 3D shape. A few approaches to 3D extended target tracking involved measurement models using basic geometric shapes [22] or restricted shape estimation [23,24,25]. The methods are suitable for when the detailed shape information is not critical for the application. For instance, where the target’s shape is unknown beforehand, random matrix models can represent the target using forms like ellipsoid, or rectangular cuboids. However, the representation power of RM models remains limited to these relatively simple geometric shapes. To address more complex geometries, Gaussian Processes have been developed for 3D space, allowing the modeling of arbitrary shaped star-convex targets [26,27]. While GPs effectively capture complex 3D shapes, their representation (often via a radius function) typically results in a high-dimensional state vector. This high dimensionality poses challenges for filtering algorithms and increases the computational burden. In contrast, the double Fourier series (DFS) shape model proposed in [28] utilizes a shape vector of significantly lower dimensionality compared to GP models, while also enabling the estimation of both shape and pose. It’s worth noting that a common characteristic across many of these 3D shape measurement models is the modeling of the shape-characterizing state as a random variable, typically assumed to follow a Gaussian distribution.

Differing from traditional 3D methods that rely on prior shape evolution models, our approach models the parameters defining the extent state directly, treating them as quantities with clear physical meaning. Within the Expectation Conditional Maximization (ECM) framework, we propose an extended target tracking method for the joint estimation of kinematics, extent, and orientation in 3D space. The main contributions are listed as follows.

(1) The unknown but fixed 3D shape is represented via a radial function in spherical coordinates, with a Double Fourier Series (DFS) expansion employed for its modeling. This approach converts the continuous radial function into a parametric representation characterized by a finite set of coefficients.
(2) The axis-angle representation constitutes a compact and singularity-free approach for parameterizing 3D orientation. It describes a rotation by specifying a unit vector, which defines the axis of rotation, and a scalar angle, which denotes the magnitude of rotation about this axis. Furthermore, this method exhibits both geometric intuitiveness and notable computational efficiency.
(3) Joint estimation of the target’s kinematic state and shape parameters is achieved via the Expectation Conditional Maximization (ECM) framework. The E-step infers the kinematic state using an unscented Kalman smoother filter, while the M-step estimates shape and rotation parameters by minimizing separate cost functions with added regularization for robustness and smoothness.

The remainder of this paper is organized as follows. Section 2 formulates the problem and presents the system, including the motion model, shape representation, and measurement model. Section 3 derives an ECM-based algorithm for 3D ETT problem. Section 4 presents simulation results to validate the proposed approach across two distinct scenarios. Finally, Section 5 summarizes the main findings and concludes the article.

Notation: Throughout this paper, the superscripts “−1” and “⊤” represent the inverse and transpose of a matrix, respectively; N(μ,∑) represents the Gaussian distribution with mean μ and covariance ∑; E[⋅] and E[⋅|⋅] is mathematical expectation and conditional expectation. The notation ⊗ refers to the Kronecker product. I denote the identity matrix with proper dimensions; |⋅| and Tr[⋅] denote the determinant and trace of matrices, respectively; $||\cdot |{|}_{2}$ denotes the Euclidean norm.

## 2. Problem Formulation

This section first introduces a model for the spatial extent of 3D target, which employs a radial function parameterized via a Double Fourier Series (DFS). The target’s orientation is then described using the axis–angle representation, a method characterized by its compactness and singularity-free nature. Following this, two kinematic state evolution models specifically designed for 3D extended target tracking are presented. Finally, the paper details the measurement model that integrates this DFS-based shape representation with the selected axis–angle orientation.

### 2.1. DFS-Based Shape Model for 3D Target

Advanced sensors, such as depth cameras and LiDAR, can generate detailed three-dimensional (3D) point cloud data, extracting precise shape estimates from such data is crucial for object classification and predicting future behavior. To effectively learn shapes from these point clouds, it is essential to formulate a suitable object extent description method that possesses high representational power for diverse 3D shapes while also being sufficiently compact to support efficient online tracking algorithms.

This section details the modeling of the target shape in spherical coordinates via a radial function f(θ,φ). Such functions relate angular coordinate pairs (θ,φ) to a radial distance in each orientation and are adept at representing star-convex geometries. In our methodology, this radial function is modeled using a Double Fourier Series (DFS) expansion, the specific form of which is given by:

(1) $$\begin{array}{cc} f(\zeta ,\theta ,\varphi ) & =\zeta_{0}+\sum_{n=1}^{N}\sum_{m=1}^{M}[\zeta_{nm}^{cc}\cos(n\theta )\cos(m\phi )+\zeta_{nm}^{ss}\sin(n\theta )\sin(m\phi )\\ & +\zeta_{nm}^{sc}\sin(n\theta )\cos(m\phi )+\zeta_{nm}^{cs}\cos(n\theta )\sin(m\phi )]\\ = & S(\theta ,\varphi )\cdot \zeta \end{array}$$

where

$$\zeta =[\zeta_{0},\zeta_{11}^{cc},\zeta_{11}^{ss},\zeta_{11}^{sc},\zeta_{11}^{cs},\zeta_{12}^{cc},\zeta_{12}^{ss},\zeta_{12}^{sc},\zeta_{12}^{cs},\ldots ,\zeta_{NM}^{cc},\zeta_{NM}^{ss},\zeta_{NM}^{sc},\zeta_{NM}^{cs}]$$

$$\begin{array}{ll} S(\theta ,\varphi )=[1, & \cos\theta \cos\phi ,\sin\theta \sin\phi ,\sin\theta \cos\phi ,\\ & \cos\theta \sin\phi ,\cos\theta \cos2\phi ,\sin\theta \sin2\phi ,\sin\theta \cos2\phi ,\cos\theta \sin2\phi \ldots ,\\ & \cos N\theta \cos M\phi ,\sin N\theta \sin M\phi ,\sin N\theta \cos M\phi ,\cos N\theta \sin M\phi ] \end{array}$$

Here, $\zeta_{0}$ is the base radial parameter, $\zeta_{nm}^{cc},\zeta_{nm}^{ss},\zeta_{nm}^{sc},\zeta_{nm}^{cs}$ are the Fourier coefficients, and N,M are the number of angle θ and ϕ. θ∈[−π,π] is azimuth and ϕ∈[−π/2,π/2] is elevation. The shape parameter ζ collects $\zeta_{0}$ and all 4NM coefficients, resulting in a dimension of $d_{\zeta }=1+4NM$.

### 2.2. Orientation Representation (Axis-Angle)

To represent the target’s three-dimensional (3D) orientation, this work utilizes the axis-angle parameterization, often expressed compactly as an orientation vector ω=γa. In this formulation, a is a unit vector denoting the axis of orientation, and γ is the scalar angle defining the magnitude of rotation orientation according to the right-hand rule.

This choice of representation is underpinned by several compelling advantages pertinent to tracking applications. Firstly, the axis–angle representation (particularly in its three-parameter rotation vector form ω) provides a geometrically intuitive and unique mapping for any 3D rotation (up to 2π periodicity in angle), without requiring the satisfaction of complex inter-parameter constraints for its validity—unlike, for instance, the nine-parameter rotation matrix which must adhere to strict orthogonality and determinant conditions. Secondly, the practical application and conversion of this representation are significantly facilitated by Rodrigues’ rotation formula. This formula not only provides a straightforward and computationally efficient method for determining the effect of the rotation on a vector (i.e., rotating a vector) but also, more fundamentally, offers an explicit algorithm to compute the exponential map from the Lie algebra so (3) (the space of 3 × 3 skew-symmetric matrices, representing infinitesimal rotations) to the Special Orthogonal group SO (3) (the Lie group of finite 3D rotations) [29]. The corresponding rotation matrix R(ω) is computed using Rodrigues’ rotation formula:

(2) $$R(\omega )=I_{3}+\sin(\gamma ){\left[b\right]}_{\times }+(1-\cos(\gamma )){\left[b\right]}_{\times }^{2}$$

where

(3) $${\left[b\right]}_{\times }=\left(\begin{array}{ccc} 0 & -b_{3} & b_{2}\\ b_{3} & 0 & -b_{1}\\ -b_{2} & b_{1} & 0 \end{array}\right)$$

${\left[b\right]}_{\times }$ is the skew-symmetric cross-product matrix of b. The axis-angle coordinates ω=γa are a natural and compact rotation representation in terms of its geometric building blocks.

### 2.3. Kinematic State Model

A. line kinematic model

The kinematic state $x_{t}={\left[c_{t}^{⊤}\;v_{t}^{⊤}\right]}^{⊤}$ at time t includes the center position $c_{t}=\left[c_{x,t}\;c_{y,t}\;c_{z,t}\right]$ and velocity components $v_{t}=[v_{x,t}\;v_{y,t}\;v_{z,t}]$. The dynamic model of extended target is modeled by

(4) $$x_{t+1}=Fx_{t}+w_{t}$$

where F is the state transition matrix, the process noise $w_{t}\sim N(0,C)$.

For constant velocity model (CV) [27]:

(5) $$F=\left[\begin{array}{cc} 1 & T\\ 0 & 1 \end{array}\right]⊗I_{3}\;\;\;\;C=\left[\begin{array}{cc} \frac{T^{3}}{3} & \frac{T^{2}}{2}\\ \frac{T^{2}}{2} & T \end{array}\right]\;⊗(\sigma_{c}^{2}I_{3})$$

where T is sampling time, $\sigma_{c}$ is the process noise variance for the centroid, ⊗ is the Kronecker product, and $I_{3}$ denotes an identity matrix.

B. nonlinear kinematic model

While the target moves with a constant acceleration (CA) between time step, the acceleration state vector $a_{t}=[a_{x,t}{\;a}_{y,t}{\;a}_{z,t}]$ is augmented the kinematic state. Combining these into the 9-dimensional state vector $x_{t}={\left[c_{t}^{⊤},v_{t}^{⊤},a_{t}^{⊤}\right]}^{⊤}$, the state evolution model is given as follows [30],

(6) $$x_{t+1}=F^{CA}x_{t}+w_{t}^{CA}$$

where $F^{CA}$ is the state transition matrix, the process noise $w_{t}^{CA}\sim N(0,Q^{CA})$.

(7) $$F^{CA}=\left[\begin{array}{ccc} I_{3} & TI_{3} & \frac{1}{2}T^{2}I_{3}\\ 0_{3} & I_{3} & TI_{3}\;\\ 0_{3} & 0_{3} & I_{3} \end{array}\right]\;Q^{CA}=\sigma_{a}^{2}\left[\begin{array}{ccc} \frac{T^{5}}{20}I_{3} & \frac{T^{4}}{8}I_{3} & \frac{T^{3}}{6}I_{3}\\ \frac{T^{4}}{8}I_{3} & \frac{T^{3}}{3}I_{3} & \frac{T^{2}}{20}I_{3}\;\\ \frac{T^{3}}{6}I_{3} & \frac{T^{2}}{2}I_{3} & TI_{3} \end{array}\right]$$

The scalar $\sigma_{a}^{2}$ represents the intensity of the underlying noise process and is a crucial tuning parameter for the CA model.

### 2.4. Measurement Model

Sensor data at time t consists of a set of $N_{t}$ noisy 3D measurements ${\left\{z_{j,t}\right\}}_{j=1}^{N_{t}}$ originating from the target’s surface. Each measurement $z_{j,t}$ is related to the state and parameters via the non-line measurement function plus noise. A single measurement can be expressed as

(8) $$z_{j,t}=c_{t}+f_{local}+e_{t}$$

where $c_{t}$ is the center of the target, $f_{local}$ is the radial function representation which describes the target’s shape in local coordinate. However, the measurement $z_{j,t}$ is obtained in global coordinates. Consequently, a global-to-local transformation is essential. This transformation yields the following local representation,

(9) $$f_{local}=R_{global}^{local}f_{global}=R(\omega )\left[\begin{array}{l} f(\zeta ,\theta_{j,t},\phi_{j,t})\cos(\phi_{j,t})\cos(\theta_{j,t})\\ f(\zeta ,\theta_{j,t},\phi_{j,t})\cos(\phi_{j,t})\sin(\theta_{j,t})\\ f(\zeta ,\theta_{j,t},\phi_{j,t})\sin(\phi_{j,t}) \end{array}\right]$$

$f(\zeta ,\theta_{j,t},\phi_{j,t})$ represents the radial function form in global coordinate, which modeled using DFS parameterized by the coefficient ζ, and $e_{t}$ is zero-mean Gaussian measurement noise with covariance $R_{t}$. $\left(\theta_{j,t},\phi_{j,t}\right)$ is the angle pair of the measurement source on the target surface that originates $z_{j,t}$.

R(ω) is the rotation matrix that converts the global coordinates to local coordinates for the corresponding orientation parameter ω. This parameters ω utilize the axis-angle representation, expressed as ω=γa, where $a=[a_{x},a_{y},a_{z}]$ is a unit vector ($||a|{|}_{2}=1$) indicating the axis of rotation orientation, and γ is the angle of rotation about the axis a (typically in radial, following the right-hand rule). This parameter ω compactly stores both axis and angle information. Specifically, its magnitude $||\omega |{|}_{2}=\gamma$ give the rotation angle, while its direction $\omega \;/||\omega |{|}_{2}=a$ give the axis for rotation orientation (for γ≠0). This axis-angle representation uses only 3 parameters (the components of ω) and advantageously avoids the gimbal lock singularities associated with Euler angles.

The parameter ζ is the set of coefficients for the DFS that defines the entire 3D shape of the target. The core assumption of the model is that the target is a rigid body. Therefore, this single, unified shape parameter ζ is used to calculate the surface radius for any point $\left(\theta_{t},\phi_{t}\right)$ on the object’s surface. When process the jth measurement, and we use its specific projected angles $\left(\theta_{j,t},\phi_{j,t}\right)$ as inputs to the universal shape function defined by ζ to get the predicted radius at that exact spot.

Let $Z^{G}=z_{j,t}-c_{t}$ and $\left(\theta_{j,t},\phi_{j,t}\right)$ be expressed as,

(10) $$\theta_{j,t}=\arccos(\frac{x_{t}^{G}}{||z_{j,t}-c_{t}|{|}_{2}})$$

(11) $$\phi_{j,t}=\mathrm{atan}2(y_{t}^{G},x_{t}^{G})$$

where $Z_{t}^{G}≜(x_{t}^{G},y_{t}^{G},z_{t}^{G})$.

As the measurement $z_{j,t}$ cannot be isolated as an explicit function of the state vector and noise, Equation (8) defines an implicit measurement model. It can be rewritten as:

(12) $$\begin{array}{ll} 0= & \underset{h(x_{t},z_{j,t},\zeta ,\omega )}{\underset{︸}{-z_{j,t}+c_{t}+R(\omega )\left[\begin{array}{l} f(\zeta ,\theta_{j,t},\phi_{j,t})\cos(\phi_{j,t})\cos(\theta_{j,t})\\ f(\zeta ,\theta_{j,t},\phi_{j,t})\cos(\phi_{j,t})\sin(\theta_{j,t})\\ f(\zeta ,\theta_{j,t},\phi_{j,t})\sin(\phi_{j,t}) \end{array}\right]}}+e_{t}\\ & =h(x_{t},z_{j,t},\zeta ,\omega )+e_{t} \end{array}$$

The zero vector appearing in Equation (12) can be interpreted as representing a pseudo-measurement. This pseudo-measurement is effectively a nonlinear function of the state and the original measurement, assumed to be corrupted by additive Gaussian noise.

## 3. ECM Tracking Algorithm Based on DFS

This section details the DFS-ECM framework for joint motion state, shape, and orientation estimation. Its core, the Expectation Conditional Maximization (ECM) algorithm, uses a batch method involving iterative Kalman smoothing over a sliding data window. This incorporates information from previous and subsequent scans. For a batch window from time 1 to $T_{b}$, the ECM framework defines these variables:

$$X=(x_{1},\ldots ,x_{t}\ldots ,x_{T_{b}})$$

$$Z=(Z_{1},\ldots ,Z_{t}\ldots ,Z_{T_{b}})$$

$$\Lambda =(\Lambda_{1},\ldots ,\Lambda_{t}\ldots ,\Lambda_{T_{b}})$$

where X denotes the latent variables within the sliding window; Z represents the set of measurements within the sliding window, including $Z_{t}=(z_{1,t},\ldots z_{j,t},\ldots ,z_{N_{t},t})$; Λ denotes the complete sequence of unknown parameters, where each $\Lambda_{t}=(\omega ,\zeta )$ of the sliding window at time step t. ECM is an iterative optimization method whose objective is to maximize the log-likelihood function of the incomplete data (latent variable) X with respect to the parameter Λ.

The main terms used in this algorithm can be found in Table 1.

We define the complete-data log-likelihood function $L(\Lambda^{\left(i+1\right)})$ and its corresponding conditional expectation function $Q(\Lambda^{\left(i+1\right)};\Lambda^{\left(i\right)})$ of the (i+1)th iteration as follows:

(13) $$L(\Lambda^{\left(i+1\right)})=\ln p(X^{\left(i+1\right)},Z|\Lambda^{\left(i+1\right)})$$

(14) $$Q(\Lambda^{\left(i+1\right)};\Lambda^{\left(i\right)})=E[L(\Lambda^{\left(i+1\right)})|Z,\Lambda^{\left(i\right)}]$$

As the Q function involves multiple unknown parameters, a direct application of the traditional maximization step is not feasible. Instead, we utilize multiple conditional maximization steps to iteratively estimate the parameters.

### 3.1. E-Step

The primary purpose of the E-step is to estimate the state $x_{t}$ and its corresponding error covariance matrix $P_{t}$, a process that involves an Unscented Kalman forward filter (UKF) [31,32] and backward Rauch–Tung–Striebel Smoother smoother [33,34]. The forward propagation is performed as,

Let n be the dimension of the kinematic state $x_{t}$, we generate 2n+1 sigma points $X_{k,t-1}$ based on the previous state estimate $x_{t-1}$ and error covariance $P_{t-1}$ (state initialized as $x_{0}=\mu_{0}$, $P_{0}=\sum_{0}$):

(15) $$X_{k,t-1}=\left\{\begin{array}{ll} x_{t-1} & \;k=0\\ x_{t}+{(\sqrt{(n+\lambda )P_{t-1}})}_{k} & k=1,\ldots ,n\\ x_{t}-{(\sqrt{(n+\lambda )P_{t-1}})}_{k} & k=n+1,\ldots ,2n \end{array}\right.$$

The weights for mean $W_{k}^{\left(m\right)}$ and covariance $W_{k}^{\left(c\right)}$ are:

(16) $$W_{k}^{\left(m\right)}=\left\{\begin{array}{l} \frac{\lambda }{n+\lambda }\;\;k=0\\ \frac{1}{2(n+\lambda )}\;\;k\neq 0 \end{array}\right.$$

(17) $$W_{k}^{\left(c\right)}=\left\{\begin{array}{l} \frac{\lambda }{n+\lambda }+\;(1-\alpha^{2}+\beta )\;\;\;k=0\\ \frac{1}{2(n+\lambda )}\;\;k\neq 0 \end{array}\right.$$

where $\lambda =\alpha^{2}(n+\kappa )-n$ is a scaling parameter, α controls the distance of the Sigma points from mean, κ is a secondary parameter β is used to incorporate covariance information.

Prediction step of UKF:

(1) Propagate Sigma Points: Pass each state sigma point through the process model F,

(18) $$X_{k,t|t-1}=FX_{k,t-1}$$

(2) Compute the predicted state ${\hat{x}}_{t|t-1}$ and predict error covariance $P_{t|t-1}$:

(19) $${\hat{x}}_{t|t-1}=\sum_{k=0}^{2n}W_{k}^{\left(m\right)}X_{k,t|t-1}$$

(20) $$P_{t|t-1}=\sum_{k=0}^{2n}W_{k}^{\left(c\right)}(X_{k,t|t-1}-{\hat{x}}_{t|t-1}){\left(X_{k,t|t-1}-{\hat{x}}_{t|t-1}\right)}^{⊤}+C$$

Update step of UKF: the update proceeds sequentially for each measurement. Initialize the updated state and error covariance for this time step with predicted values,

(21) $${\hat{x}}_{0,t}={\hat{x}}_{t|t-1}$$

(22) $$P_{0,t}=P_{t|t-1}$$

the measurements ${\left\{z_{j,t}\right\}}_{j=1}^{N_{t}}$ are processed using the following steps,

(1) Predict the jth measurement $Z_{j,k,t|t-1}$ for each sigma point $X_{k,t|t-1}$ using the parameter ζ,ω,

(23) $$Z_{j,k,t|t-1}=h(X_{k,t|t-1},\zeta ,\omega )$$

(2) Compute the jth weighted average measurement ${\hat{z}}_{j,t|t-1}$ of the predicted sigma point,

(24) $${\hat{z}}_{j,t|t-1}=\sum_{k=0}^{2n}W_{k}^{\left(m\right)}Z_{k,t|t-1}$$

(3) Estimate the jth innovation covariance $P_{j,zz,t|t-1}$ and the cross-covariance $P_{j,xz,t|t-1}$,

(25) $$P_{j,zz,t|t-1}=\sum_{k=0}^{2n}W_{k}^{\left(c\right)}(Z_{k,t|t-1}-{\hat{z}}_{j,t|t-1}){\left(Z_{k,t|t-1}-{\hat{z}}_{j,t|t-1}\right)}^{⊤}+R_{t}$$

(26) $$P_{j,xz,t|t-1}=\sum_{k=0}^{2n}W_{k}^{\left(c\right)}(X_{k,t|t-1}-{\hat{x}}_{t|t-1}){\left(Z_{k,t|t-1}-{\hat{z}}_{j,t|t-1}\right)}^{⊤}$$

(4) Calculate the jth Kalman Gain $K_{j,t}$,

(27) $$K_{j,t}=P_{j,xz,t|t-1}P_{j,zz,t|t-1}^{-1}$$

(5) Update the jth state ${\hat{x}}_{j,t}$ and its error covariance $P_{j,t}$,

(28) $${\hat{x}}_{j,t}={\hat{x}}_{j-1,t}+K_{j,t}(0-{\hat{z}}_{j,t|t-1})$$

(29) $$P_{j,t}=P_{j-1,t}-K_{j,t}P_{j,zz,t|t-1}K_{j,t}^{⊤}$$

When processing all $N_{t}$ measurements, the UKF state ${\hat{x}}_{t}$ estimation and its error covariance $P_{t}$ are:

(30) $${\hat{x}}_{t}={\hat{x}}_{N_{t},t}$$

(31) $$P_{t}=P_{N_{t},t}$$

After sequentially processing the data from time t=1 to $t=T_{b}$ with an Unscented Kalman Filter (UKF), we obtain the series of filter states ${\left\{{\hat{x}}_{t}\right\}}_{t=1}^{T_{b}}$ and error covariances ${\left\{P_{t}\right\}}_{t=1}^{T_{b}}$. Subsequently, the Rauch–Tung–Striebel smoothing algorithm is applied to compute the smoothed state ${\hat{x}}_{t|T_{b}}$ and its error covariance $P_{t|T_{b}}$ as follows,

The backward pass begins at the final time step $T_{b}$. (1) Initialize the smoothed state ${\hat{x}}_{0,t|T_{b}}$ and error covariance $P_{0,t|T_{b}}$ for this time step with UKF estimation,

(32) $${\hat{x}}_{0,t|T_{b}}={\hat{x}}_{T_{b}}$$

(33) $$P_{0,t|T_{b}}=P_{T_{b}}$$

The algorithm then iterates backward in time, from $t=T_{b}-1$ down to t=1. For each time step t, the following computations with the measurements ${\left\{z_{j,t}\right\}}_{j=1}^{N_{t}}$ are performed:

(2) Compute the jth Smoothed Gain $G_{j,t}$,

(34) $$G_{j,t}=P_{j,xz,t+1|t}P_{t+1|t}^{-1}$$

where $P_{j,xz,t+1|t}$ is the jth cross covariance and $P_{t+1|t}$ is the predict error covariance of forward UKF, both obtained from the forward UKF pass.

(3) Compute the jth Smoothed state ${\hat{x}}_{j,t|T_{b}}$,

(35) $${\hat{x}}_{j,t|T_{b}}={\hat{x}}_{j-1,t|T_{b}}+G_{t}({\hat{x}}_{t+1|T_{b}}-{\hat{x}}_{t+1|t})$$

(4) Compute the jth Smoothed error Covariance $P_{j,t|T_{b}}$,

(36) $$P_{j,t|T_{b}}=P_{j-1,t|T_{b}}+G_{j,t}(P_{t+1|T_{b}}-P_{t+1|t})G_{j,t}^{⊤}$$

When processing all $N_{t}$ measurements, the smoothed state ${\hat{x}}_{t|T_{b}}$ estimation and its error covariance $P_{t|T_{b}}$ are,

(37) $${\hat{x}}_{t|T_{b}}={\hat{x}}_{N_{t},t|T_{b}}$$

(38) $$P_{t|T_{b}}=P_{N_{t},t|T_{b}}$$

Considering the Markov property of state propagation and the independence of measurement sources, the log-likelihood function $L(\Lambda^{\left(i+1\right)})$ is written as,

(39) $$L(\Lambda^{\left(i+1\right)})=\ln p(x_{0})+\sum_{t=1}^{T_{b}}\ln p(x_{t+1}|x_{t})+\sum_{t=1}^{T_{b}}\sum_{j=1}^{N_{t}}\ln p(z_{j,t}|x_{t},\Lambda^{\left(i+1\right)})$$

So,

(40) $$\begin{array}{cc} Q(\Lambda^{\left(i+1\right)};\Lambda^{\left(i\right)})) & =E_{Z,\Lambda^{\left(i\right)}}[\ln p(x_{0})]+\sum_{t=1}^{T_{b}}E_{Z,\Lambda^{\left(i\right)}}\ln p(x_{t}|x_{t-1})+\sum_{t=1}^{T_{b}}\sum_{j=1}^{N_{t}}E_{Z,\Lambda^{\left(i\right)}}\ln p(z_{j,t}|x_{t},\Lambda^{\left(i+1\right)})\\ & =\sum_{t=1}^{T_{b}}\sum_{j=1}^{N_{t}}E_{Z,\Lambda^{\left(i\right)}}\ln p(z_{j,t}|x_{t},\Lambda^{\left(i+1\right)})+Const \end{array}$$

Under the assumption of Gaussian measurement noise, the measurement likelihood becomes,

(41) $$p(z_{j,t}|x_{t},\omega ,\zeta )=N(z_{j,t}-h(x_{t},z_{j,t},\omega ,\zeta );0,R_{t})$$

Insert Equation (41) into (40), Q is,

(42) $$\begin{array}{l} Q(\Lambda^{\left(i+1\right)};\Lambda^{\left(i\right)})==\sum_{t=1}^{T_{b}}\sum_{j=1}^{N_{t}}E_{Z,\Lambda^{\left(i\right)}}\ln p(z_{j,t}|x_{t},\Lambda_{t}^{\left(i+1\right)})+Const\\ =-\frac{1}{2}\sum_{j=1}^{N_{t}}\{\sum_{t=1}^{T_{b}}\ln|R_{j,t}|+\mathrm{Tr}\sum_{t=1}^{T_{b}}\left(R_{j,t})E_{Z,\Lambda^{\left(i\right)}}[(z_{j,t}-h(x_{t},z_{j,t},\omega ,\zeta )){\left(•\right)}^{⊤}\right]\}+Const \end{array}$$

where (•) denotes the term same as the former one.

### 3.2. M-Step

After obtaining $Q(\Lambda^{\left(i+1\right)};\Lambda^{\left(i\right)})$ in the E-step of the algorithm, the task of the M-step is to find $\Lambda^{\left(i+1\right)}$, thereby updating the target’s shape and axis-angle parameters from the previous iteration. Since $Q(\Lambda^{\left(i+1\right)};\Lambda^{\left(i\right)})$ is a non-linear function of the parameters, its analytical solution is generally difficult to obtain. Here, parameters are estimated by minimizing regularized cost function which equivalent to maxing $Q(\Lambda^{\left(i+1\right)};\Lambda^{\left(i\right)})$ estimation.

(1) Shape parameter optimization

The shape parameter ζ are estimated by minimizing the cost function J(ζ) over a sliding window of $T_{b}$ time steps (t=1 to $T_{b}$).

(43) $$J_{original}(\zeta )=(\frac{1}{\sum_{t=1}^{T_{b}}N_{t}}\sum_{t=1}^{T_{b}}\sum_{j=1}^{N_{t}}||z_{j,t}-h({\hat{x}}_{t},z_{j,t},\zeta ,\hat{\omega })|{|}_{R_{t}^{-1}}^{2})+\eta_{\zeta }||\zeta_{2:end}|{|}_{2}^{2}+P_{s}(\zeta )$$

where, ${\hat{x}}_{t}$, $\hat{\omega }$ are the estimates of state and orientation at time t within the window.$||e|{|}_{R^{-1}}^{2}=e^{⊤}R^{-1}e$ denotes the squared Mahalanobis distance. The first term is the average squared Mahalanobis distance over all valid measurements in the window. $\eta_{\zeta }$ is the L2 regularization parameter applied to the coefficients (excluding $\eta_{0}$) to prevent overfitting and improving conditioning. $P_{s}(\zeta )$ is the smoothness penalty term.

**Remark** **1.** *Using the original cost function $J_{original}(\zeta )$ can lead to artificial bulges or distortions near the poles (ϕ=± π/2). This occurs because the spherical coordinate system used by the DFS is degenerate at the poles, and measurement near these regions can exert disproportionate influence during optimization, leading to overfitting localized at the poles.*

To mitigate these artifacts, we introduce a latitude-dependent weight factor $w_{pole}(\phi )$ that down-weights measurements near the poles. A common choice is

(44) $$w_{pole}(\phi )=\cos^{h}(|\phi |)$$

where h≥1 (e.g., h=1 or h=2) is a tuning parameter controlling the strength of the suppression.

The modified cost function J(ζ) is,

(45) $$J(\zeta )=(\frac{1}{\sum_{t=1}^{T_{b}}\sum_{j=1}^{N_{t}}w_{pole}({\hat{\phi }}_{j,t})}\sum_{t=1}^{T_{b}}\sum_{j=1}^{N_{t}}w_{pole}({\hat{\phi }}_{j,t})||z_{j,t}-h({\hat{x}}_{t},z_{j,t},\zeta ,\hat{\omega })|{|}_{R_{t}^{-1}}^{2})+\eta_{\zeta }||\zeta |{|}_{2}^{2}+P_{s}(\zeta )$$

**Remark** **2.** By multiplying the squared Mahalanobis distance of each measurement by $w_{pole}(\phi )$, we reduces the contribution of measurements where |ϕ| is close to π/2. This is mathematically equivalent to assuming a higher effective measurement noise variance ($R_{j,t}^{*}=R_{t}/w_{pole}({\hat{\phi }}_{j,t})$) near the poles. It prevents the optimizer from overly fitting to potentially unreliable projections in these geometrically sensitive regions. Allowing the regularization terms to enforce a smoother, more plausible shape. The tuning parameter h control how aggressively measurements near the poles are down-weighted.

Improved Smoothness Penalty: A physically meaningful smoothness penalty for the DFS on a sphere penalizes higher spatial frequencies more heavily. The indices n and m in the DFS correspond to the frequencies in the azimuth and elevation directions, respectively. Higher values of n and m represent finer details or oscillations on the surface. A suitable penalty terms $P_{s}(\zeta )$ is given by:

(46) $$P_{s}(\zeta )=\eta_{s}\sum_{n=1}^{N}\sum_{m=1}^{M}\left(n^{\nu }+m^{\nu }\right)({\left(\zeta_{n,m}^{cc}\right)}^{2}+{\left(\zeta_{n,m}^{ss}\right)}^{2}+{\left(\zeta_{n,m}^{sc}\right)}^{2}+{\left(\zeta_{n,m}^{cs}\right)}^{2})$$

where $\eta_{s}$ is the smoothness regularization strength parameter. The term ($n^{\nu }+m^{\nu }$) acts as a weighting factor that increases with the frequency indices n and m. A common is ν=2, making the penalty proportional to ($n^{2}+m^{2}$). The sum of squares involves all four coefficient types for the given (n, m) pair.

(2) Orientation parameter optimization

Minimize J(ω) at step t, regularizing towards the previous estimate $\hat{\omega }$:

(47) $$J(\omega )=\frac{1}{N}\sum_{j=1}^{N_{t}}||z_{j,t}-h({\hat{x}}_{t},z_{j,t},\hat{\zeta },\omega )|{|}_{R_{t}^{-1}}^{2}+\eta_{\omega }||\omega -\hat{\omega }{\left|\right|}_{2}^{2}$$

where ${\hat{x}}_{t},\hat{\zeta }$ are the current estimates of state and shape. $\hat{\omega }$ is the estimated orientation parameter from the previous time step. $\eta_{\omega }$ is the regularization parameter controlling the penalty for deviating from the previous orientation estimation, promoting temporal smoothness.

(3) Optimization Constraints

Constraints can be applied during optimization:

 A. Shape parameter constraints:

$\zeta_{0}>\varepsilon$ (small positive value)

 B. Rotation parameter constraints:

angle limit: $||\omega |{|}_{2}\leq \pi$.

Iteration of the EM algorithm ceases upon convergence or reaching a limit. Convergence is typically determined when the change in the likelihood function value between successive iterations is sufficiently small. Alternatively, termination occurs if the iteration count reaches a predefined upper bound.

The overall algorithm flow is summarized in Algorithm 1.
**Algorithm 1.** DFS-ECM **Initial parameters:** Measurement set batch Z, State batch X, orientation and extent parameters Λ, Maximum Iterations I
**begin**
  Setup i=0
1       **while** not converged **and**
i<I
**do**
2              **expectation:**
3                  **for**
$t=1:T_{b}$
**do**
4                       **for**
$j=1:N_{t}$
**do**
5                           Calculate ${\hat{x}}_{j,t}$ and $P_{j,t}$ according to (28–29) 
6                        **end for**
7                    **end for**
8                    **for**
$t=T_{b}:-1:1$
**do**
9                        Calculate smoothed ${\hat{x}}_{t|T_{b}}$ and $P_{t|T_{b}}$ according to (37–38) 
10                    **end for**

11               **maximization:**
12                   **Calculate**
ζ, ω according to (45,47)
13               i++;
14         **end while**
**end begin**

## 4. Simulation Results

Our proposed method, referred to as DFS-ECM in this section, are evaluated in two distinct scenarios. The simulations are run on an Intel Core™ i7-7700 processor at 3.6 GHz with 32 GB of RAM using MATLAB 2021a. Root Mean Square Error (RMSE) and the Intersection-Over-Union (IOU) are used to assess overall performance.

### 4.1. Evaluation Index

Root-Mean-Square Error (RMSE) is defined below:

(48) $$RMSE_{c}=\sqrt{\frac{1}{M}\sum_{i=1}^{M}{\left(C_{i}-c_{i}\right)}^{2}}$$

where $RMSE_{c}$ represents the RMSE of the parameter c, $C_{i}$ represents the true and $c_{i}$ represents the estimated value.

The shape estimation performance is evaluated by Intersection-Over-Union (IOU). IOU is defined as follows:

(49) $$IOU(v_{true},\hat{v})=\frac{volume(v_{true}\cap \hat{v})}{volume(v_{true}\cup \hat{v})}$$

where volumes $v_{true}$ is the true target shape, and $\hat{v}$ represents the estimate. $IOU(v_{true},\hat{v})\in [0,1]$, 1 corresponds to a perfect match, while 0 indicates no intersection between the true and the estimated extent.

### 4.2. Scenario I

**Figure 1:**
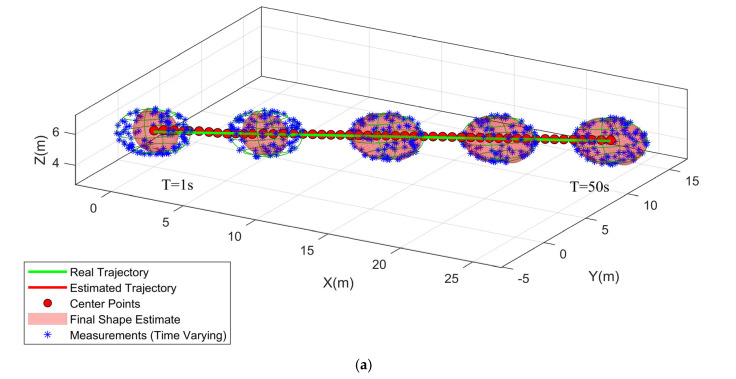

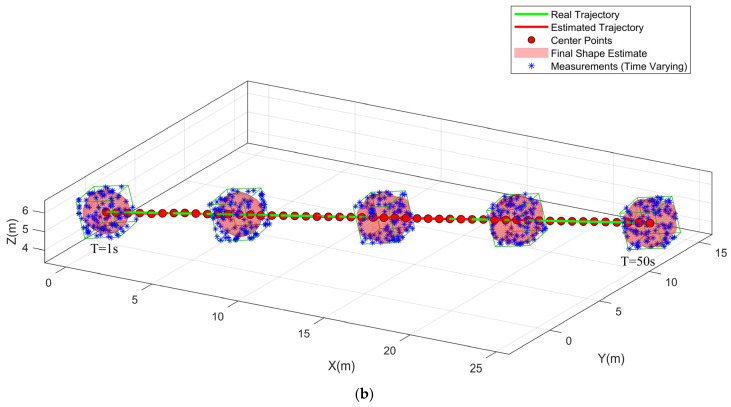
(**a**) ellipsoid target tracking; (**b**) cubic target tracking.

In this scenario, we test the DFS-ECM algorithm with ellipsoid and cubic target. Every target moves in a linear trajectory with the constant velocity (CV) model, and with a sensor located at the origin. The trajectory of target motion is presented in Figure 1. The initial kinematic state is set to [0 m, 0 m, 0 m, 10 m/s, 10 m/s, 0 m/s]. At each instant, 100 points measurements are originated from random sources which are sample from a uniform distribution defined over the target surface. The sampling time of all algorithms is set to 1 s. The orientation is represented by the axis angle parameter ω=(0,0,0).

The process noise standard deviations are set to $\sigma_{c}=0.1$, For the sigma points, a scaling parameter α=1, κ=0, β=2. For the extent parameter, $\zeta_{0}=1.0$ and $\zeta_{2,\ldots ,d_{\zeta }}=0$, the covariance matrix is set to $diag(0.1,\ldots ,0.1)\in {\mathbb{R}}^{d_{\zeta }\times d_{\zeta }}$. The degree of double Fourier series is chosen as M=N=2.

**Figure 2:**
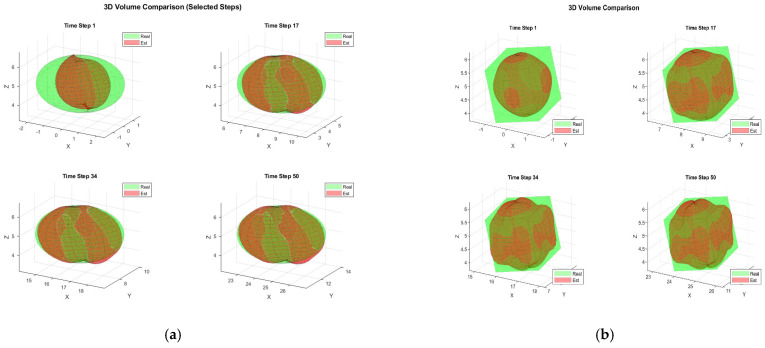
Target construction, (**a**) ellipsoid target, (**b**) cube target.

**Figure 3:**
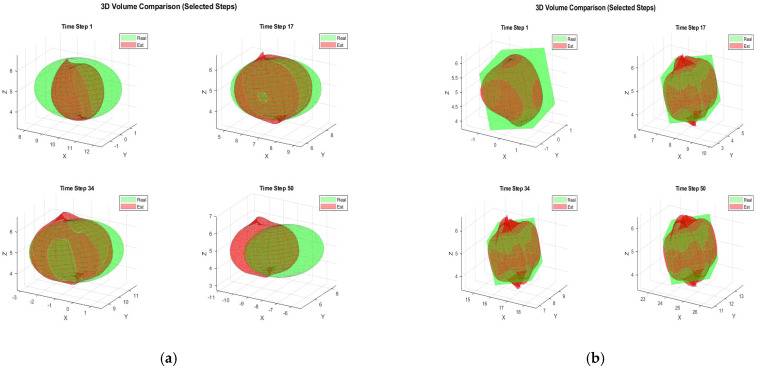
Target construction without weight (**a**) ellipsoid target (**b**) ellipsoid target.

Figure 2 illustrates the typical results and tracking trajectories in one Monte Carlo run. Figure 2a,b depict the tracking scene for the ellipsoid, cube, respectively. For enhanced clarity regarding the reconstruction outcomes, Figure 3 illustrates the reconstruction results for 3D shapes obtained without incorporating the weight. A comparison between Figure 2 and Figure 3 reveals that optimizing shape reconstruction with the weight yields superior accuracy.

**Figure 4:**
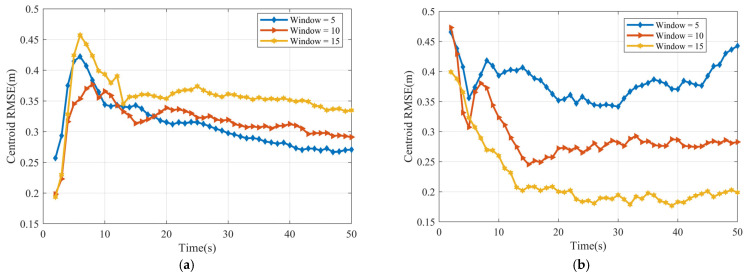
Centroid RMSE (**a**) ellipsoid target RMSE (**b**) cube target RMSE.

We evaluated centroid estimation for linearly moving ellipsoidal and cubic targets using 100 Monte Carlo trials (randomizing noise and sample points). Figure 4 shows the centroid RMSE for sliding window lengths $T_{b}=5$, 10, and 15. For the ellipsoid (Figure 4a), $T_{b}=5$ yields the minimum RMSE; performance worsens for $T_{b}=10$ and $T_{b}=15$, suggesting potential overfitting. For the cube (Figure 4b), $T_{b}=15$ is optimal. Generally, errors decrease over the simulation (indicating stabilization), and the relative performance of different window sizes remains consistent.

**Figure 5:**
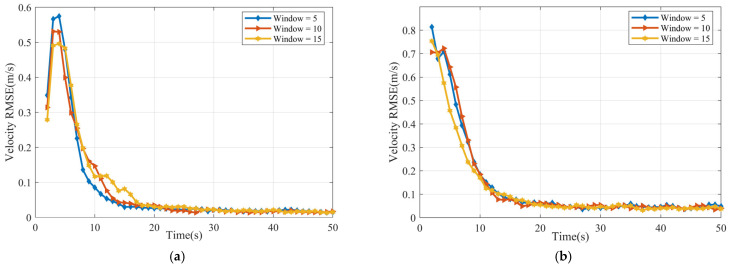
Velocity RMSE (**a**) ellipsoid target RMSE (**b**) cube target RMSE.

Figure 5 illustrates the RMSE values of the velocity estimates with the sliding window length $T_{b}=5$, 10, and 15. This indicates that while there’s a large initial uncertainty in velocity, the estimation becomes highly accurate over time, and the choice of window size (within this range) has minimal impact on the long-term velocity estimation accuracy.

**Figure 6:**
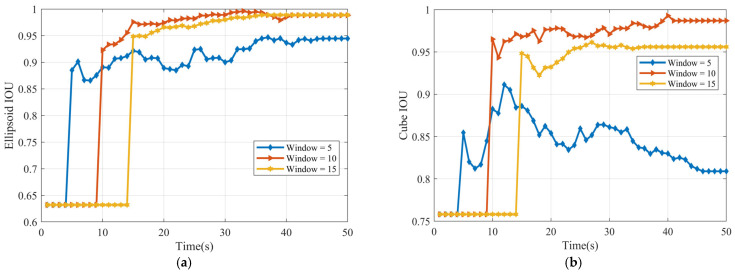
IOU (**a**) ellipsoid target IOU (**b**) cube target IOU.

Figure 6 presents the Intersection over Union (IOU) values for the shape estimates, obtained using sliding window lengths of $T_{b}=5$, 10, and 15. For both the ellipsoidal and cubic targets, the analysis indicates that the highest IOU score (representing the best shape estimate accuracy) is achieved with the window length $T_{b}=15$.

As detailed in Table 2, the errors associated with the estimated orientataion parameter components remain consistently below 0.1 for both ellipsoidal and cubic target types, indicating a high degree of estimation accuracy.

For comparison, we use a standard random matrix-based extended target tracker [35], henceforce denoted as RM. Table 3 summarizes the comparative performance of the RM and DFS-ECM approaches. The results consistently show that the DFS-ECM method outperforms the RM model in terms of centroid estimation accuracy, velocity estimation accuracy, and shape estimation quality (as measured by IOU) across all tested scenarios.

As presented in Table 4, increasing the number of measurements positively impacts overall tracking performance. Specifically, a higher measurement count leads to a lower Centroid RMSE, indicating improved positioning accuracy. Concurrently, the shape estimation quality, measured by IOU, steadily improves for both the ellipsoid and the cube, confirming that more data yields more accurate reconstructions.

### 4.3. Scenario II

**Figure 7:**
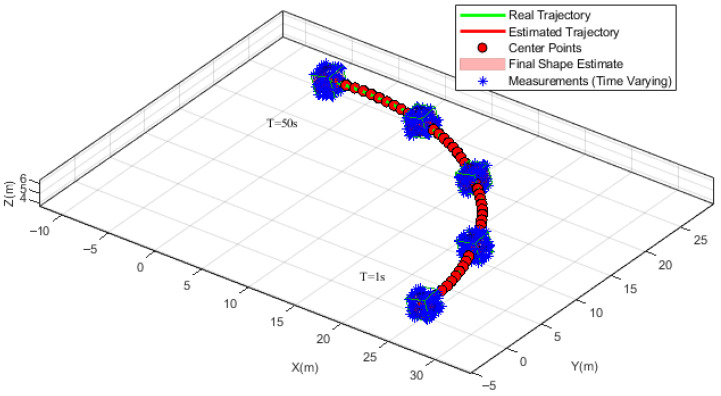
Cube targe tracking.

The performance of the DFS-ECM algorithm is further evaluated using a nonlinear scenario. In this experiment, a cubic target moves along a curved trajectory in three-dimensional space, as depicted in Figure 7. The target’s motion is governed by the constant acceleration (CA) model, which is defined in Equation (6). This simulation utilizes the same DFS model hyperparameters and EM parameters as those employed in Scenario Ⅰ. Within the CA model, the scalar parameter $\sigma_{a}^{2}$ is set to 0.001. At each instant, 100 measurement points are sampled uniformly from the target’s surface. The orientation is represented by the axis angle parameter ω=(0.1,−0.2,0.05).

**Figure 8:**
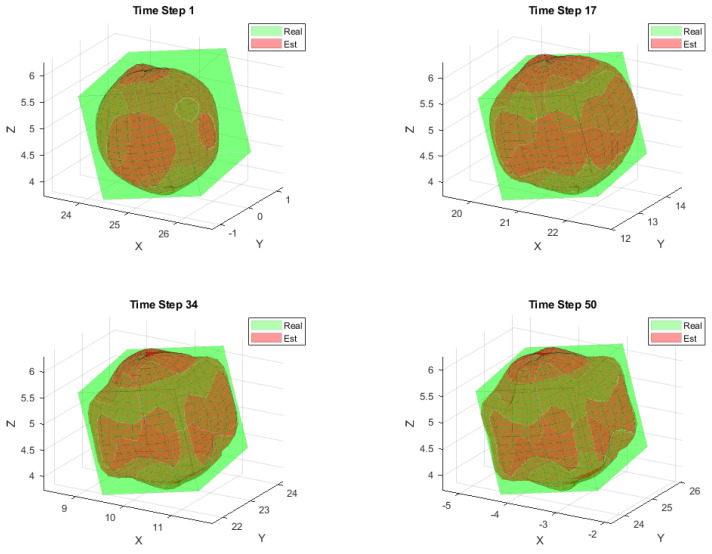
Cube target.

To better illustrate the reconstruction quality, Figure 8 presents magnified views of typical reconstruction examples for the cubic target. These examples demonstrate satisfactory reconstruction performance.

**Figure 9:**
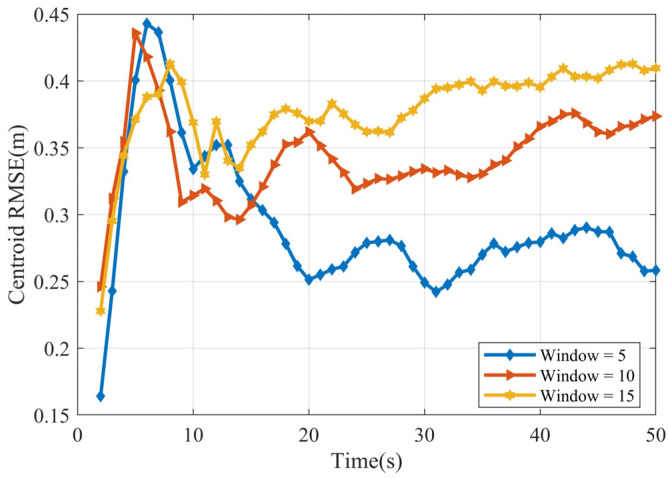
Centroid RMSE of cube.

Figure 9 presents the centroid RMSE over time for the cubic target, comparing window lengths $T_{b}=5$, 10, and 15. Optimal performance (lowest RMSE) is achieved with the shortest window,$T_{b}=5$. Conversely, the larger window lengths ($T_{b}=10$ and $T_{b}=15$) yield higher errors, potentially indicating underfitting in this nonlinear scenario. However, all three RMSE curves exhibit convergence towards stable values over time.

**Figure 10:**
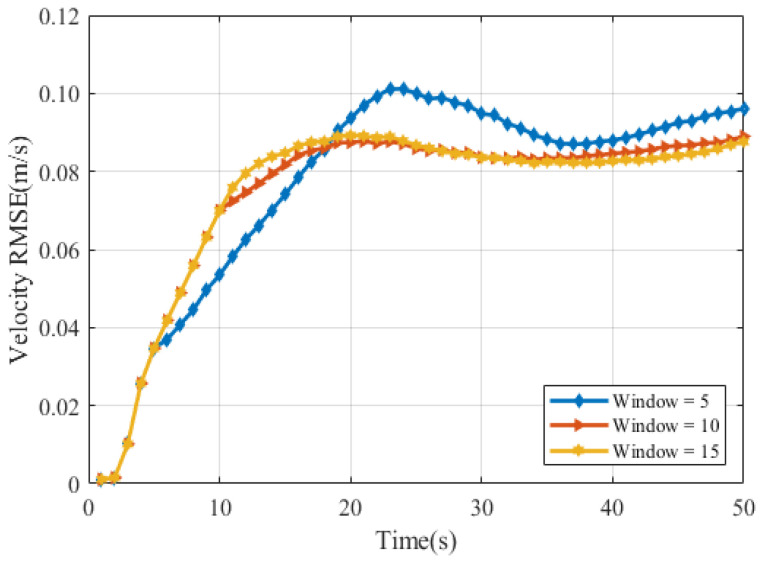
Velocity RMSE of cube.

The velocity RMSE for the cubic target is presented in Figure 10. Notably, while the larger window sizes ($T_{b}=10$ and $T_{b}=15$) show similar performance with lower peak errors and better long-term stability (lower RMSE), the smallest window ($T_{b}=5$) suffers from a higher peak error (occurring later) and maintains a higher error level beyond approximately 18 s.

**Figure 11:**
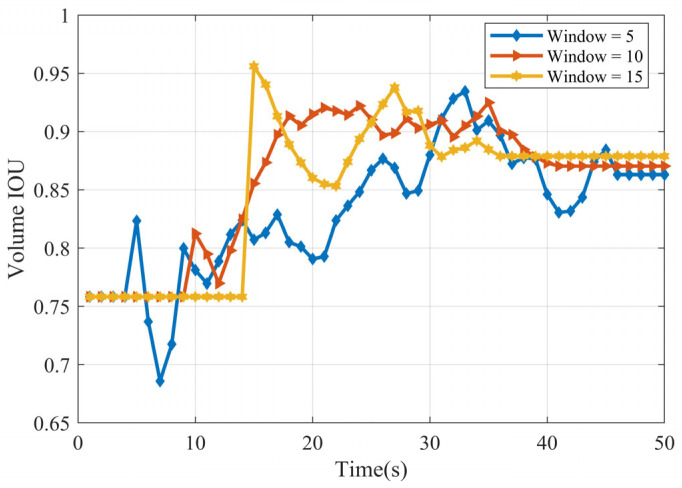
IOU of cube.

Figure 11 plots the volume Intersection over Union (IOU), representing shape estimation accuracy, over time for three different window sizes. Towards the end of the observation period (after 40 s), the performance stabilizes for all window sizes. They converge to similar and relatively high IOU values, approximately in the range of 0.86–0.88. While these results are strong, the accuracy is slightly below that achieved for the cube in the linear scenario. We attribute this minor performance difference to the inherent challenges of the nonlinear model. The nonlinearity likely introduces small errors into the centroid position estimate, which subsequently impacts the overall accuracy of the shape reconstruction.

**Figure 12:**
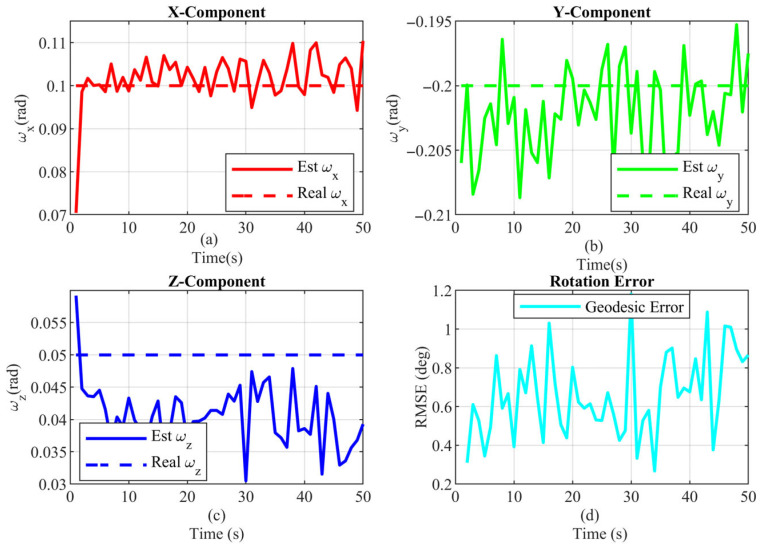
Rotation parameters estimate (**a**) rotation about the x-axis (**b**) rotation about the y-axis (**c**) rotation about the z-axis (**d**) orientation RMSE.

The performance of orientation parameter estimation is detailed in Figure 12. The error dynamics for individual rotation components within a single Monte Carlo run are illustrated in Figure 12a–c. The overall accuracy, depicted by the average Root Mean Square Error (RMSE) across all Monte Carlo runs, is shown in Figure 12d. The low error values demonstrate the algorithm’s capability for accurate angle estimation.

**Figure 13:**
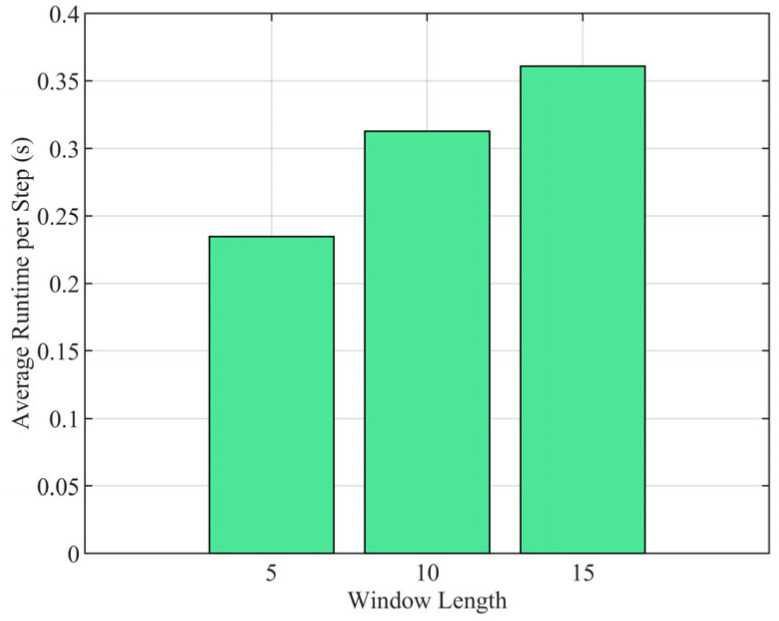
Average runtime.

Figure 13 illustrates the computational cost, specifically the average processing time per step required by the algorithm, as a function of different window lengths ($T_{b}=5$, 10, and 15). A clear positive correlation is evident: longer window lengths necessitate incNreased computation time per step.

## 5. Conclusions

This paper proposed a method for joint 3D extended object tracking with unknown but fixed shape in the framework of Expectation Conditional Maximization (ECM). By introducing a shape representation based on a Double Fourier Series (DFS) radial function and utilizing the axis-angle representation for orientation, this method enables the direct estimation of target shape and orientation parameters, overcoming the reliance on prior shape evolution models. Leveraging the ECM algorithm, kinematic state inference is performed using an Unscented Kalman Filter (UKF) in the E-step, while shape and orientation parameters are estimated via minimization of regularized cost functions in the M-step. This ultimately achieves the joint, robust, and smooth estimation of kinematics, shape, and orientation. The methods provide a full expression for the target’s shape, this detailed information can be used in 3D applications such as navigation, identification and classification. Future work will focus on adaptive tuning and joint estimation schemes to further advance the capability of 3D target tracking.

## Figures and Tables

**Table 1:** Summary of the main terms used in the derivation of the algorithm.

$P_{t}$	The error covariance of target state at time t
$P_{t|t-1}$	The predict error covariance of target state at time t
$P_{j,zz,t|t-1}$	The jth innovation covariance at time t
$P_{j,xz,t|t-1}$	The jth cross-covariance at time t
$P_{j,t}$	The jth error covariance at time t
$P_{j,t|T_{b}}$	The jth smoothed covariance at time t
$P_{t|T_{b}}$	The smoothed error covariance at time t
$p(x_{t}|x_{t-1})$	Transition Probability density of state $x_{t}$
$p(z_{j,t}|x_{t},\Lambda^{\left(i\right)})$	The likelihood function
$p(X^{\left(i+1\right)},Z|\Lambda^{\left(i\right)})$	The likelihood function of complete data
$p(x_{0})$	The prior Probability density of state
$P_{s}(\zeta )$	The smoothness penalty term of the cost function
i	Iteration times of ECM
j	Index of measurement number

**Table 2:** Orientation parameters estimation.

Target	Angle	T = 5 (rad)	T = 10 (rad)	T = 15 (rad)
Ellipsoid	$\omega_{x}$	0.020	0.030	0.030
	$\omega_{y}$	0.080	0.050	0.060
	$\omega_{z}$	0.050	0.050	0.050
Cube	$\omega_{x}$	0.043	0.042	0.043
	$\omega_{y}$	0.004	0.004	0.003
	$\omega_{z}$	0.015	0.012	0.016

**Table 3:** Performance comparation of two diffenernt algorithm.

Target	Algorithm	Centroid RMSE (m)	Velocity RMSE (m/s)	IOU
Ellipsoid	RM	0.27	0.10	0.90
	DFS-ECM	0.26	0.07	0.96
Cube	RM	0.27	0.10	0.70
	DFS-ECM	0.20	0.07	0.95

**Table 4:** Performance comparation of different muber of measurements.

Target	Number of Measurement	Centroid RMSE (m)	IOU
Ellipsoid	10	0.29	0.91
	20	0.28	0.92
	40	0.27	0.96
Cube	10	0.30	0.90
	20	0.28	0.92
	40	0.288	0.95

## Data Availability

The data presented in this study are available upon request from the corresponding authors.
